# Complete chloroplast genome of *Meconopsis integrifolia* (Papaveraceae)

**DOI:** 10.1080/23802359.2019.1698353

**Published:** 2019-12-11

**Authors:** Rui Li, Xianye Ma, Xiaofeng Zhang, Dangwei Zhou, Huan Wang, Tingfeng Cheng, Wenjuan Wang

**Affiliations:** a College of Medicine, Xi’an International University, Xi’an, Shaanxi, China;; b College of Ecological Environment and Resources, Qinghai Nationalities University, Xining, Qinghai, China;; c Key Laboratory of Adaptation and Evolution of Plateau Biota (AEPB), Northwest Institute of Plateau Biology, Chinese Academy of Sciences, Xining, Qinghai, China

**Keywords:** *Meconopsis integrifolia*, Tibetan medicine, chloroplast genome, phylogenetic analysis

## Abstract

*Meconopsis integrifolia* (Maxim.) Franch is a traditional Tibetan medicinal material. In this study, we sequenced and assembled the complete chloroplast genome of *M. integrifolia*. The chloroplast genome is 152,714 bp in length, containing a pair of inverted repeated (IR) region of 25,627 bp that are separated by a large single copy (LSC) region of 83,706 bp, and a small single copy (SSC) region of 17,754 bp. Moreover, a total of 126 functional genes were annotated, including 86 protein-coding genes, 37 tRNA genes, and 8 rRNA genes. In the maximum likelihood phylogenetic tree, *M. integrifolia* clustered closely with three *Papaver* species.

*Meconopsis integrifolia* is the endemic species belonging to the poppy family Papaveraceae, consisting of 43 species (Wu and Chuang 1980). It is a potential horticulture plant as it has a big and beautiful yellow flower. Hence, it attracts botanists and horticulturalists all over the world. Moreover, as traditional Tibetan medicine, it has many important active compounds, which exhibit anti-inflammatory and analgesic activities (Guo et al. 2016). Chloroplast (cp) DNA sequences are the most important source of genetic markers to study distribution of paternal genes and paternally based molecular phylogenetic relationships (Shaw et al. 2007). Moreover, the cp transformation had high level of expression, lack of post-transcription gene silencing, site-specific transformation, and environmental friendliness (Maliga 2004; Bock 2007), which would promote its usage in agriculture and pharmaceutical industry. Here, the complete cp genome (accession number: MN453867) of *M. integrifolia* was *de novo* sequenced with Illumina sequencing platform 2500 (San Diego, CA).

In this study, fresh leaves of *M. integrifolia* were collected from Guoluo (N 34.70°, E 99.00°; Alt. 4200 m), Qinghai, China. The voucher specimens were kept in Herbarium of the Northwest Institute of Plateau Biology, Chinese Academy of Sciences (HNWP, DWZhou0121). Total DNA was extracted from the fresh leaves with the DNeasy Plant MiniKit (Qiagen, Carlsbad, CA) according to the manufacturer’s instructions. The next experiment and analysis scheme referred to Wang et al. (2019). DNA with good integrity and purity was used for library construction and sequencing with the Illumina Hiseq 2500 (San Diego, CA). Approximately 9.78 GB of clean data were yielded. The trimmed reads were mainly assembled by SPAdes (Bankevich et al. 2012). The assembled genome was annotated using CpGAVAS (Liu et al. 2012).

The complete cp genome of *M. integrifolia* is 152,714 bp in length, containing a pair of inverted repeated (IRa and IRb) regions of 25,627 bp, each, that are separated by a large single copy (LSC) region of 83,706 bp, and a small single copy (SSC) region of 17,754 bp. Moreover, a total of 126 functional genes were annotated, including 86 protein-coding genes, 37 tRNA genes, and 8 rRNA genes. The protein-coding genes, tRNA genes, and rRNA genes account for 71.67, 27.21, and 6.34% of all annotated functional genes, respectively.

Analyses based on the complete cp genomes and concatenated sequences of 67 common protein-coding genes among the studied species were conducted. The two sets of sequences were aligned by MAFFT (Katoh and Standley 2013), and then the alignments were adjusted by the Gblocks program (Castresana 2000). The maximum-likelihood (ML) method was employed to construct phylogenetic trees by RAxML version 8.0 software using the GTRGAMMA model. Bootstrap analysis for each branch was calculated by 500 replications. The phylogenetic tree (Figure 1) indicated that *M. integrifolia* has a closer relationship with three *Papaver* species (*Papaver somniferum*, *Papaver rhoeas*, and *Papaver orientale*). The results provide valuable information for future studies on phylogenetic and evolution adaptation on *M. integrifolia* and its related species.

**Figure 1.**
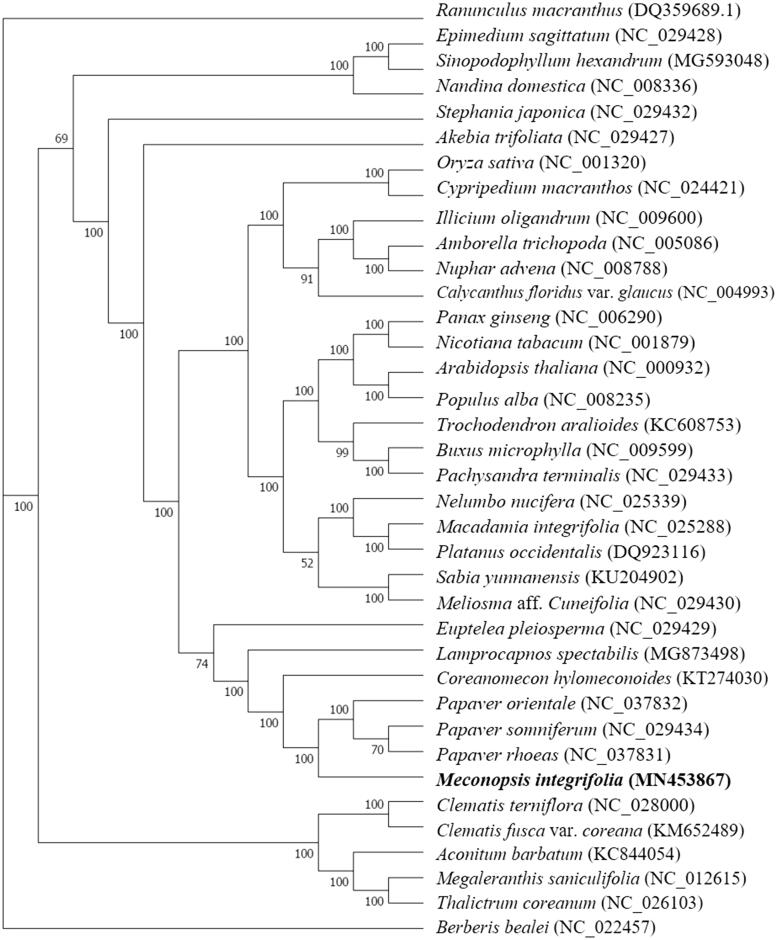
The ML tree based on chloroplast genome sequences.
